# Influencing factors of sleep disorders and sleep quality in healthcare workers during the COVID‐19 pandemic: A systematic review and meta‐analysis

**DOI:** 10.1002/nop2.1871

**Published:** 2023-06-06

**Authors:** Qian Lv, Wenguang Zhou, Yue Kong, Silu Chen, Baoling Xu, Fangfang Zhu, Xianying Shen, Zhaojun Qiu

**Affiliations:** ^1^ Teaching and Research Department 900TH Hospital of Joint Logistics Support Force Fuzhou China; ^2^ Department of Equipment Chenggong Hospital of Xiamen University (the 73th Group Military Hospital of People's Liberation Army) Xiamen China; ^3^ Teaching and Research Department Fuzong Clinical Medical College of Fujian Medical University (900TH Hospital of Joint Logistics Support Force) Fuzhou China; ^4^ Nursing College Fujian Medical University Fuzhou China; ^5^ Nursing College Fujian University of Traditional Chinese Medicine Fuzhou China

**Keywords:** COVID‐19, frontline workers, healthcare workers, insomnia, sleep disorders, sleep quality

## Abstract

**Aim:**

The aim of this study was to identify the influencing factors of sleep disorders and sleep quality in healthcare workers during the COVID‐19 pandemic.

**Design:**

Systematic review and meta‐analysis of observational research.

**Methods:**

The databases of the Cochrane Library, Web of Science, PubMed, Embase, SinoMed database, CNKI, Wanfang Data, and VIP were systematically searched. The quality of studies was assessed using the Agency for Healthcare Research and Quality evaluation criteria and the Newcastle–Ottawa scale.

**Results:**

A total of 29 studies were included, of which 20 were cross‐sectional studies, eight were cohort studies, and 1 was a case–control study; 17 influencing factors were finally identified. Greater risk of sleep disturbance was associated with female gender, single relationship status, chronic disease, insomnia history, less exercise, lack of social support, frontline work, days served in frontline work, department of service, night shift, years of work experience, anxiety, depression, stress, received psychological assistance, worried about being infected, and degree of fear with COVID‐19.

**Conclusions:**

During the COVID‐19 pandemic, healthcare workers did have worse sleep quality than the general population. The influencing factors of sleep disorders and sleep quality in healthcare workers are multifaceted. Identification and timely intervention of resolvable influencing factors are particularly important for preventing sleep disorders and improving sleep.

**Patient or Public Contribution:**

This is a meta‐analysis of previously published studies so there was no patient or public contribution.

## 1 INTRODUCTION

In December 2019, a novel coronavirus pneumonia pandemic broke out in Wuhan, China, which was named coronavirus disease 2019 (COVID‐19) by the World Health Organization (WHO, 2020) on January 12, 2020 (Jin et al., 2020) and declared a public health emergency of international concern on the January 30, 2020 (Cucinotta & Vanelli, 2020). Presently, respiratory droplet transmission and contact transmission are considered the main transmission routes of COVID‐19 (General Office of National Health Committee & Office of State Administration of Traditional Chinese Medicine, 2020). The age distribution of infected individuals shows that no age group is resistant to COVID‐19 (Khan et al., 2020). Close contact with COVID‐19 patients and asymptomatic individuals carrying the virus is associated with high risk of contracting the infection (Wang, Hu, et al., 2020). Healthcare workers have to be in close contact with COVID‐19 patients when treating and caring for them, which increases their risk of infection (Kluytmans‐van den Bergh et al., 2020). According to WHO data, as of April 3, 2022, there have been more than 489 million cases worldwide and more than 6.1 million deaths, establishing this disease as a serious threat to human health. As the number of infections increases and thousands of patients require urgent care, the burden on the global healthcare system increases; consequently, healthcare workers are overworked, and their sleep and mental health are severely affected; this is particularly true for frontline healthcare workers directly attending to COVID‐19 patients or suspected infected persons (Jiang et al., 2021).

## 2 BACKGROUND

Sleep is essential for the regulation of psychological and physiological processes. Sleep disorders and poor sleep quality harm physical and mental health, impair physical and mental recovery, and reduce immunity (Zielinski et al., 2016). These issues leave healthcare workers vulnerable to COVID‐19. A systematic review of the sleep quality of healthcare workers and patients during the COVID‐19 pandemic showed that the sleep quality of frontline healthcare workers was significantly poorer than that of the general population (Lin et al., 2021). Another cross‐sectional study analysed the psychological conditions of 7071 healthcare workers in 24 provinces in China. The incidence rates of anxiety, depression, and sleep disorders among healthcare workers during the COVID‐19 pandemic were significantly higher during the first 2–5 months of the COVID‐19 pandemic than before the outbreak (35%, 36%, and 37% vs. 25%, 28%, and 26%, respectively; Luo et al., 2021). Several studies (Chen, Liu, et al., 2021; Li et al., 2020) have shown that frontline healthcare workers with anxiety and depression are more likely to develop sleep disorders. To address these issues, mental health agencies around the world have developed various psychological assistance services, including telephone, internet, and apps (Dong et al., 2021). However, targeted interventions will be difficult without a comprehensive understanding of the factors influencing sleep disorders in healthcare workers during the COVID‐19 pandemic.

Since the COVID‐19 outbreak, some scholars have studied the prevalence and influencing factors of sleep disorders and sleep quality in healthcare workers (Jahrami et al., 2022; Salari et al., 2020). However, the influencing factors included in the analysis of many studies were not comprehensive, and the research conclusions were not consistent; therefore, the quality of the research needs to be further evaluated. Thus, this study used a meta‐analysis approach to further explore the influencing factors of sleep disorders and sleep quality in healthcare workers during the COVID‐19 pandemic. The findings would serve as high‐quality evidence to enable more targeted prevention or intervention measures in clinical practice to help healthcare workers avoid and resolve sleep disorders.

## 3 DESIGN

This systematic review and meta‐analysis was conducted according to the Preferred Reporting Items for Systematic Reviews and Meta Analyses (PRISMA) statement (Page et al., 2021) guidelines. The research protocol was registered in the “International Prospective Register of Systematic Review” (PROSPERO) in 2022 (CRD 42022359478, available from: link https://www.crd.york.ac.uk/PROSPERO/).

## 4 ETHICS

This study generated evidence to identify influencing factors of sleep disorders and sleep quality in healthcare workers during the COVID‐19 pandemic. Data used were obtained from previously published studies, and therefore, research ethics committee approval was not required.

## 5 METHODS

### 5.1 Search strategy

We searched the following databases for studies published between November 2019 and December 2022: Cochrane Library, Web of Science, PubMed, Embase, SinoMed database, China National Knowledge Infrastructure (CNKI), Wanfang Data, China Science Journal Database (VIP). MeSH terms used included “sleep quality” OR “sleep disorders” OR “insomnia” OR “sleep deprivation” OR “sleep” OR “sleep hygiene” OR “dyssomnias” AND “healthcare workers” OR “medical workers” OR “HCWS” OR “health personnel” OR “healthcare providers” OR “nursing staff” OR “staff” OR “Physicians” AND “influencing factor” OR “risk factor” OR “related factor” OR “predictor” OR “high risk factor” OR “risk factor” AND “cross sectional study” OR “cross sectional survey” OR “case control study” OR “case control” OR “cohort study” OR “survey.”

### 5.2 Inclusion and exclusion criteria

From among the results obtained using the abovementioned keywords, we included the following: (1) studies conducted during the COVID‐19 epidemic; (2) surveys administered to healthcare workers, particularly frontline healthcare workers (who have direct contact with confirmed or suspected COVID‐19 patients); (3) studies that clearly put forward the reasons or factors that affected the sleep disorders and sleep quality of healthcare workers during the COVID‐19 pandemic; (4) studies designed as cross‐sectional studies, cohort studies, or case–control studies; (5) studies with their language of publication being Chinese or English. We excluded (1) duplicate publications, (2) studies with abstracts but no full texts, and (3) studies scoring <8 points and <7 points on the Agency for Healthcare Research and Quality scale (Rostom et al., 2004) and the Newcastle–Ottawa scale (Stang, 2010), respectively, indicated inadequate study quality.

### 5.3 Data extraction

Two researchers independently screened the studies and extracted data according to the inclusion and exclusion criteria. Any disagreements were resolved by consulting a third party. A self‐designed standardized data extraction table was used, which included the first author, publication year, survey site, study type, survey object, sample size, number of frontline healthcare workers, survey tools, and influencing factors and their odds ratio (OR) values and 95% confidence interval.

### 5.4 Quality appraisal

The quality of the cross‐sectional studies was evaluated using the Agency for Healthcare Research and Quality evaluation criteria, which included study limitations, directness of intervention, consistency and precision of implementation, reporting bias, and risk of bias, with a total of 11 points: 0–3 points, 4–7 points, and 8–11 points indicative of low, medium, and high quality of the study, respectively. Cross‐sectional studies with a score of ≥8 points were included in this study. The quality of case–control studies and cohort studies was evaluated using the Newcastle–Ottawa scale, which included selected population, comparability of groups, and assessment of either the exposure or outcome of interest for studies, with a total of nine points: 0–4 points, 5–6 points, and 7–9 points indicated low, medium, and high quality of the study, respectively. Studies with a score of ≥7 points were included in this study. Specific scores can be used to evaluate the quality of the studies.

### 5.5 Statistical synthesis and analysis

Descriptive statistics were used to summarize the characteristics of the final included studies. We used OR and 95% CI as the effect size of each influencing factor. Meta‐analysis was performed using the R language meta and dmetar packages. The heterogeneity was determined using Cochran's *Q* chi‐squared test. If *p* was ≥0.10 and *I* ^2^ was ≤50%, homogeneity was indicated and the fixed‐effects model was used; otherwise, the random‐effects model was used. Sensitivity analysis was performed by comparing the consistency of the results of the two models. Publication bias was assessed using Egger's test and funnel plot. *p* < 0.05 was considered to be statistically significant.

## 6 RESULTS

### 6.1 Study selection

**FIGURE 1:**
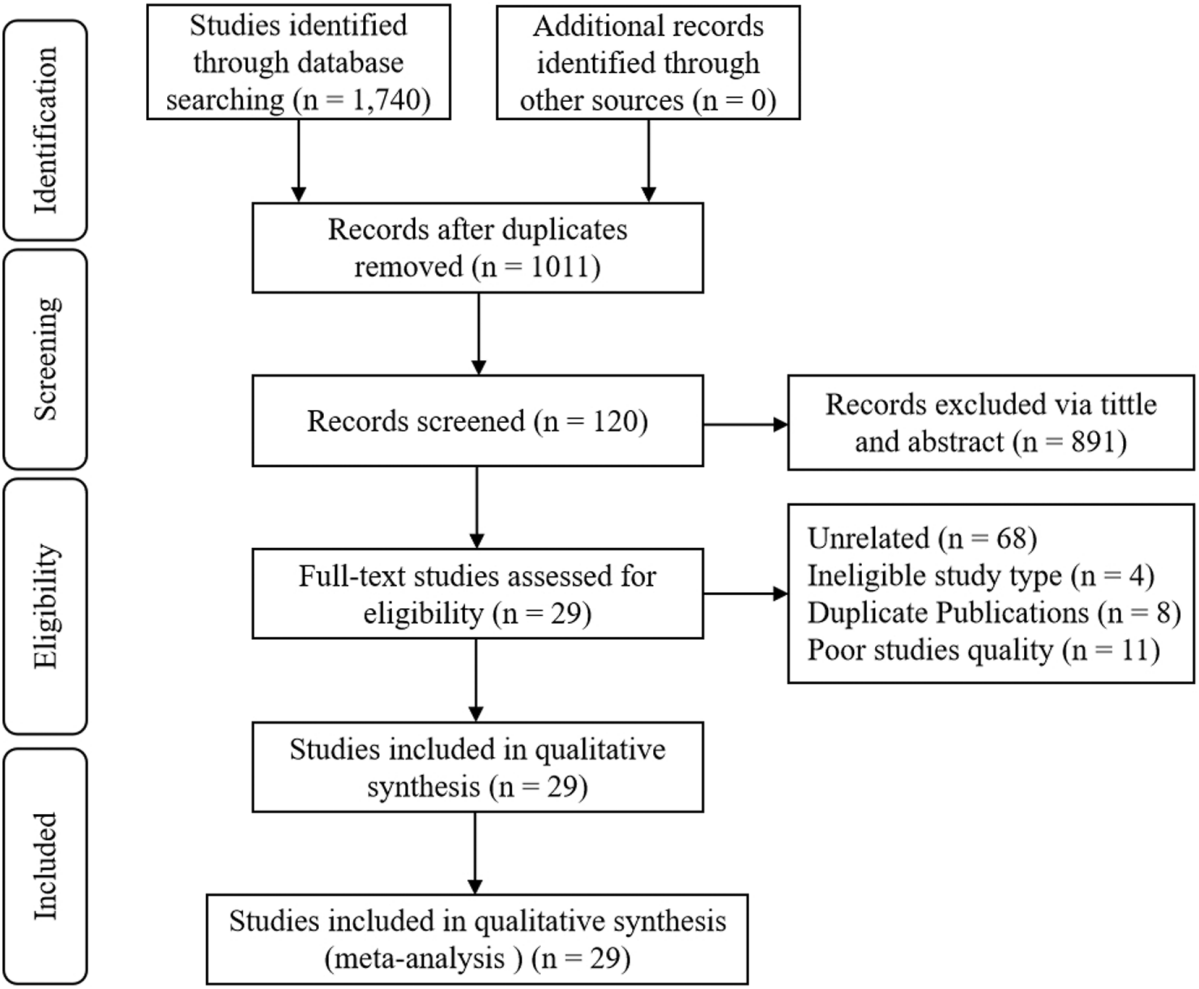
Research screening flow chart.

The researchers initially searched a total of 1740 studies in Chinese and English databases. Among these, 729 were excluded due to duplicate publications. Two researchers then independently screened the remaining studies (*n* = 1011) according to the inclusion and exclusion criteria; the first author evaluated 891 studies as ineligible; the second author evaluated 886 studies as ineligible. To reach a consensus, the third author participated in the discussion, and finally, 891 studies were considered ineligible. After reading the full text of the remaining 120 studies, 91 studies were excluded. The reasons for the exclusion were as follows: unrelated (*n* = 68); the study was identified as having poor quality (*n* = 11); the manuscript was a duplicate publication (*n* = 8); and the study was not a cross‐sectional study, case–control study, or cohort study (*n* = 4); after these exclusions, 29 studies were finally included. The screening process is shown in Figure 1.

### 6.2 Study characteristics

Among the 29 included studies, 20 were cross‐sectional studies, eight were cohort studies, and one was a case–control study; the 29 included studies encompassed 33,743 healthcare workers, including 18,181 frontline healthcare workers. The quality of the studies was high. These studies included a total of 39 influencing factors; if ≥2 studies mentioned the same influencing factor, the influencing factor was extracted. Furthermore, if the studies provided the odds ratio and 95% confidence interval of the influencing factors or the original data that can be transformed, these factors were incorporated into calculating the pooled effect size. In the end, 25 influencing factors were extracted, and 23 influencing factors contributed to the pooled effect size. The two remaining influencing factors could not be pooled because only one study could obtain data (Table 1).

**TABLE 1:** Basic characteristics of the included studies.

First author	Study design	Survey object	Sample size	Number of frontline healthcare workers	Survey tools	Influencing factors	Quality score
Gao et al. (2021)	Cross‐sectional study	Frontline nurses + non‐frontline nurses	343	99	ISI	①⑨⑮	9
Liang et al. (2021)	Cross‐sectional study	Frontline nurses + non‐frontline nurses	4237	894	ISI	①③⑪⑬⑭	9
Zhou et al. (2021)	Cross‐sectional study	Frontline nurses	2784	2784	AIS	⑤⑮⑯⑲⑳	8
Yin et al. (2021)	Cohort study	Frontline nurses + non‐frontline nurses	1128	310	PSQI	①⑬⑫⑤	8
Xu et al. (2021)	Case control study	Frontline healthcare workers	1028	1028	PSQI	①⑩⑭	7
Chen, Ma, et al. (2021)	Cross‐sectional study	Frontline healthcare workers + non‐frontline healthcare workers	504	39	PSQI	⑤⑱⑲⑳㉓	8
Pan et al. (2021)	Cross‐sectional study	Frontline healthcare workers + non‐frontline healthcare workers	482	145	ISI	⑤⑦	9
Xie et al. (2021)	Cohort study	Frontline healthcare workers	528	528	PSQI	⑭⑰	8
Cai et al. (2021)	Cross‐sectional study	Frontline nurses	504	504	AIS	⑥⑧⑨⑪	8
Wu et al. (2020)	Cross‐sectional study	Frontline nurses	106	106	PSQI	⑫⑲㉑	8
Wang, Liu, et al. (2020)	Cross‐sectional study	Frontline healthcare workers	1056	1056	ISI	①⑥⑧	8
Wei et al. (2020)	Cohort study	Frontline healthcare workers	2150	2150	PSQI	①②⑩	7
Deng et al. (2020)	Cross‐sectional study	Frontline nurses	230	230	PSQI	⑭⑱㉓㉕	8
Cai et al. (2020)	Cross‐sectional study	Frontline healthcare workers	54	54	AIS	①	8
Wang, Ren, et al. (2020)	Cohort study	Frontline healthcare workers	125	125	ISI	⑲㉒	7
Dong et al. (2020)	Cohort study	Frontline nurses	130	130	PSQI	⑫⑰㉔	7
Al Maqbali (2021)	Cross‐sectional study	Frontline nurses	987	987	PSQI	②④⑬⑲⑳㉑	9
Al Maqbali (2021)	Cross‐sectional study	Frontline nurses + non‐frontline nurses	1130	920	PSQI	②④⑤⑬⑯	9
Ali et al. (2021)	Cross‐sectional study	Frontline healthcare workers + non‐frontline healthcare workers	294	12	ISI	①④	8
Chen, Liu, et al. (2021)	Cohort study	Frontline nurses	236	236	PSQI	⑲⑳㉑	9
Dong et al. (2021)	Cross‐sectional study	Frontline healthcare workers + non‐frontline healthcare workers	236	197	ISI	⑦	8
Lai et al. (2020)	Cross‐sectional study	Frontline healthcare workers + non‐frontline healthcare workers	1257	522	ISI	⑤	8
Luo et al. (2021)	Cross‐sectional study	Frontline healthcare workers + non‐frontline healthcare workers	7071	2549	ISI	⑤㉔㉕	9
Que et al. (2020)	Cross‐sectional study	Frontline healthcare workers + non‐frontline healthcare workers	2285	NA	ISI	⑤	9
Wang, Huang, et al. (2020)	Cohort study	Frontline healthcare workers + non‐frontline healthcare workers	1045	401	ISI	③⑤⑬	7
Zhan et al. (2020)	Cross‐sectional study	Frontline nurses	1794	1794	AIS	①⑤⑨⑬⑭⑯⑱㉑	8
Zhang, Yang, et al. (2020)	Cohort study	Frontline healthcare workers + non‐frontline healthcare workers	1563	NA	ISI	③⑨⑩⑫㉓	8
Zhang et al. (2021)	Cross‐sectional study	Frontline healthcare workers	249	249	ISI	⑥⑰⑲⑳㉒	9
Zheng et al. (2021)	Cross‐sectional study	Frontline healthcare workers + non‐frontline healthcare workers	207	132	PSQI	①⑤⑩⑬⑭	9

*Note*: Influencing factors: ① gender, ② age, ③ education, ④ marital status, ⑤ frontline work, ⑥ days served in frontline work ⑦ protective equipment, ⑧ family or colleagues diagnosed with COVID‐19, ⑨ degree of fear with COVID‐19, ⑩ occupation, ⑪ professional title, ⑫ department of service, ⑬ years of work experience, ⑭ night shift, ⑮ personal health status, ⑯ chronic diseases, ⑰ insomnia history, ⑱ received psychological assistance, ⑲ anxiety, ⑳ depression, ㉑ stress, ㉒ fatigue, ㉓ worried about being infected, ㉔ social support, ㉕ exercise.

Abbreviations: AIS, Athens insomnia scale; ISI, Insomnia Severity Index; PSQI, Pittsburgh Sleep Quality Index; SQS, sleep quality scale.

### 6.3 Meta‐analysis results

#### 6.3.1 Influencing factors

A meta‐analysis of 23 influencing factors was performed. Using the fixed‐effects model, no heterogeneity was identified among the following seven influencing factors: days served in frontline work, family or colleagues diagnosed with COVID‐19, insomnia history, education, fatigue, department of service, and worried about being infected (*I* ^2^ ≤ 50, *p* ≥ 0.1). Using the random‐effects model, heterogeneity was identified in the following 16 influencing factors: frontline work, protective equipment, gender, age, marital status, anxiety, depression, received psychological assistance, stress, social support, occupation, chronic disease, degree of fear with COVID‐19, years of work experience, night shift, and exercise (*I* ^2^ > 50, *p* < 0.1).

**FIGURE 2:**
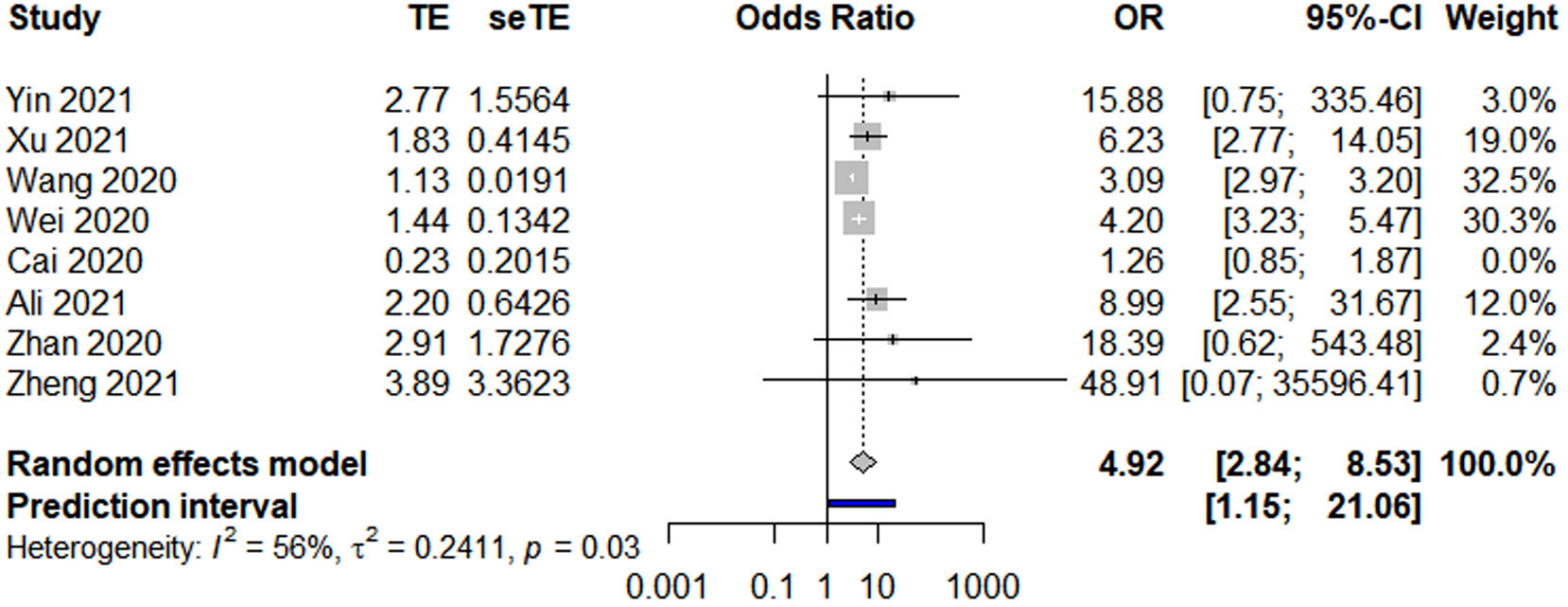
Gender forest plot.

**FIGURE 3:**
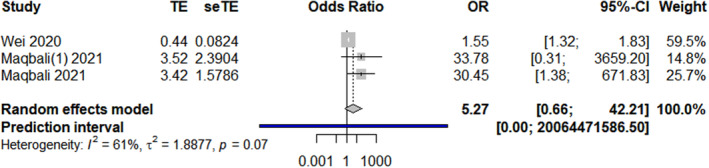
Age forest plot.

**FIGURE 4:**
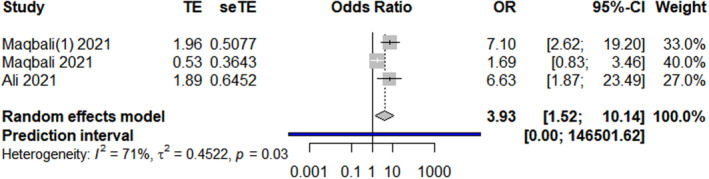
Marital status forest plot.

The results revealed that the following 17 factors affected the sleep quality and duration of healthcare workers during the COVID‐19 pandemic (*p* < 0.01, Table 2): sociodemographic factors (gender, marital status, chronic diseases, insomnia history, exercise, and social support), occupational factors (frontline work, days served in frontline work, department of service, night shift, and years of work experience), and psychological factors (anxiety, depression, stress, received psychological assistance, worried about being infected, and degree of fear with COVID‐19). Gender, age, and marital status forest plots are shown in Figures 2, 3, 4, respectively.

**TABLE 2:** Influencing factors of sleep disorders and sleep quality in healthcare workers during the COVID‐19 pandemic: a meta‐analysis.

Influencing factors	Number of studies	Heterogeneity test		Effects model	Pooled effect size	
		*I* ^2^	*p*		OR (95%CI)	*p*
Sociodemographic factors						
Gender	8 (Ali et al., 2021; Cai et al., 2020; Wang, Liu, et al., 2020; Wei et al., 2020; Xu et al., 2021; Yin et al., 2021; Zhan et al., 2020; Zheng et al., 2021)	79.2%	<0.01	Random‐effects model	4.09 (2.10, 7.99)	<0.01
Age	3 (Al Maqbali, 2021; Al Maqbali & Al Khadhuri, 2021; Wei et al., 2020)	61.5%	0.07	Random‐effects model	5.27 (0.66, 42.21)	0.12
Education	2 (Wang, Huang, et al., 2020; Zhang, Yang, et al., 2020)	46.3%	0.17	Fixed‐effects model	3.03 (0.52, 17.56)	0.22
Marital status	3 (Al Maqbali, 2021; Al Maqbali & Al Khadhuri, 2021; Ali et al., 2021)	70.6%	0.03	Random‐effects model	3.93 (1.52, 10.14)	<0.01
Chronic diseases	3 (Al Maqbali & Al Khadhuri, 2021; Zhan et al., 2020; Zhou et al., 2021)	88.2%	<0.01	Random‐effects model	3.22 (1.55, 6.69)	<0.01
Insomnia history	3 (Dong et al., 2020; Xie et al., 2021; Zhang et al., 2021)	2%	0.36	Fixed‐effects model	18.20 (3.22, 102.87)	<0.01
Exercise	2 (Deng et al., 2020; Luo et al., 2021)	87.2%	<0.01	Random‐effects model	3.25 (1.69, 6.23)	<0.01
Family or colleagues diagnosed with COVID‐19	2 (Cai et al., 2021; Wang, Liu, et al., 2020)	0	0.43	Fixed‐effects model	4.22 (0.48, 37.25)	0.19
Social support	2 (Dong et al., 2020; Luo et al., 2021)	91.3%	<0.01	Random‐effects model	2.15 (1.47, 3.14)	<0.01
Occupational factors						
Frontline work	11 (Al Maqbali & Al Khadhuri, 2021; Chen, Ma, et al., 2021; Lai et al., 2020; Luo et al., 2021; Pan et al., 2021; Que et al., 2020; Wang, Huang, et al., 2020; Yin et al., 2021; Zhan et al., 2020; Zheng et al., 2021; Zhou et al., 2021)	94.9%	<0.01	Random‐effects model	8.38 (2.87, 24.47)	<0.01
Days served in frontline work	3 (Cai et al., 2021; Wang, Liu, et al., 2020; Zhang et al., 2021)	0	0.40	Fixed‐effects model	3.84 (2.29, 6.44)	<0.01
Protective equipment	2 (Dong et al., 2021; Pan et al., 2021)	51.5%	0.15	Random‐effects model	57.89 (0.03, 104,602.84)	0.29
Occupation	4 (Wei et al., 2020; Xu et al., 2021; Zhang, Yang, et al., 2020; Zheng et al., 2021)	73%	0.01	Random‐effects model	2.51 (0.72, 8.73)	0.15
Department of service	4 (Dong et al., 2020; Wu et al., 2020; Yin et al., 2021; Zhang, Yang, et al., 2020)	0	0.90	Fixed‐effects model	6.22 (2.14, 18.12)	<0.01
Night shift	5 (Deng et al., 2020; Xie et al., 2021; Yin et al., 2021; Zhan et al., 2020; Zheng et al., 2021)	67.7%	0.01	Random‐effects model	4.63 (2.09, 10.27)	<0.01
Years of work experience	6 (Al Maqbali, 2021; Al Maqbali & Al Khadhuri, 2021; Wang, Huang, et al., 2020; Yin et al., 2021; Zhan et al., 2020; Zheng et al., 2021)	90.3%	<0.01	Random‐effects model	3.11 (1.41, 6.87)	<0.01
Psychological factors						
Anxiety	6 (Al Maqbali, 2021; Chen, Ma, et al., 2021; Wang, Ren, et al., 2020; Wu et al., 2020; Zhang et al., 2021; Zhou et al., 2021)	90.8%	<0.01	Random‐effects model	5.55 (3.06, 10.06)	<0.01
Depression	4 (Al Maqbali, 2021; Chen, Ma, et al., 2021; Zhang et al., 2021; Zhou et al., 2021)	94.5%	<0.01	Random‐effects model	12.90 (1.74, 95.79)	<0.01
Stress	3 (Al Maqbali, 2021; Wu et al., 2020; Zhan et al., 2020)	92.6%	<0.01	Random‐effects model	20.58 (2.70, 156.79)	<0.01
Received psychological assistance	3 (Chen, Ma, et al., 2021; Deng et al., 2020; Zhan et al., 2020)	89.1%	<0.01	Random‐effects model	7.63 (1.81, 31.33)	<0.01
Worried about being infected	3 (Chen, Ma, et al., 2021; Deng et al., 2020; Zhang, Yang, et al., 2020)	4.9%	0.35	Fixed‐effects model	5.94 (3.21, 10.98)	<0.01
Degree of fear with COVID‐19	3 (Cai et al., 2021; Zhan et al., 2020; Zhang, Yang, et al., 2020)	75.8%	0.02	Random‐effects model	8.18 (3.59, 18.66)	<0.01
Fatigue	2 (Wang, Ren, et al., 2020; Zhang et al., 2021)	5.9%	0.30	Fixed‐effects model	5.08 (0.35, 73.44)	0.23

#### 6.3.2 Sensitivity analysis

Sensitivity analysis of the 23 influencing factors was performed using fixed‐ and random‐effects models. The results showed that the results of random‐ and fixed‐effects model analyses were close for the following factors: frontline work, days served in frontline work, gender, marital status, anxiety, depression, received psychological assistance, stress, social support, department of service, chronic disease, degree of fear with COVID‐19, years of work experience, night shift, worried about being infected, and exercise. This finding indicated good stability of the pooled results and high reliability of the conclusion. The results for some factors (family or colleagues diagnosed with COVID‐19, protective equipment, insomnia history, age, education, fatigue, and occupation) were different, indicating that these factors were not stable for meta‐analysis (Table 3).

**TABLE 3:** Sensitivity analysis and publication bias test of influencing factors.

Influencing factors	Sensitivity analysis		Egger's test	
	Random‐effects model OR (95% CI)	Fixed‐effects model OR (95%CI)	*t*	*p*
Sociodemographic factors				
Gender	4.09 (2.10, 7.99)	3.09 (2.98, 3.21)	0.71	0.50
Age	5.27 (0.66, 42.21)	1.57 (1.34, 1.85)	5.05	0.12
Education	1.87 (1.42, 2.46)	3.03 (0.52, 17.56)	NA	NA
Marital status	3.93 (1.52, 10.14)	3.21 (1.89, 5.44)	2.00	0.30
Chronic diseases	3.22 (1.55, 6.69)	2.40 (1.86, 3.08)	8.09	0.08
Insomnia history	72.87 (0.50, 10655.48)	18.20 (3.22, 102.87)	18.42	0.03
Exercise	3.25 (1.69, 6.23)	2.65 (2.22, 3.17)	NA	NA
Family or colleagues diagnosed with COVID‐19	3.41 (3.37, 3.45)	4.22 (0.48, 37.25)	NA	NA
Social support	2.15 (1.47, 3.14)	2.57 (2.54, 2.61)	NA	NA
Occupational factors				
Frontline work	8.38 (2.87, 24.47)	2.33 (2.13, 2.56)	2.60	0.03
Days served in frontline work	3.51 (2.98, 4.12)	3.84 (2.29, 6.44)	10.24	0.06
Protective equipment	57.89 (0.03, 104602.84)	6.32 (2.48, 16.14)	NA	NA
Occupation	2.51 (0.72, 8.73)	1.76 (1.59, 1.96)	1.16	0.37
Department of service	6.05 (3.54, 10.35)	6.22 (2.14, 18.12)	4.43	0.05
Night shift	4.63 (2.09, 10.27)	2.45 (2.22, 2.69)	3.93	0.03
Years of work experience	3.11 (1.41, 6.87)	2.44 (2.09, 2.84)	0.80	0.47
Psychological factors				
Anxiety	5.55 (3.06, 10.06)	2.92 (2.85, 3.00)	3.88	0.02
Depression	12.90 (1.74, 95.79)	2.92 (2.85, 3.00)	1.74	0.22
Stress	20.58 (2.70, 156.79)	3.19 (3.08, 3.31)	1217.2	<0.01
Received psychological assistance	7.53 (1.81, 31.33)	2.28 (1.99, 2.62)	4.40	0.14
Worried about being infected	5.83 (3.74, 9.08)	5.94 (3.21, 10.98)	0.02	0.99
Degree of fear with COVID‐19	8.18 (3.59, 18.66)	7.47 (5.31, 10.51)	0.34	0.79

#### 6.3.3 Publication bias

**FIGURE 5:**
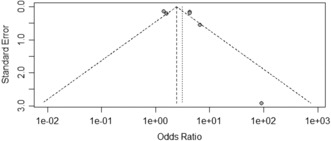
Years of work experience funnel plot.

**FIGURE 6:**
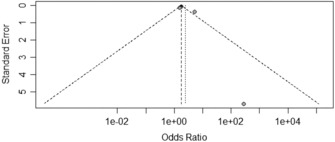
Occupation funnel plot.

**FIGURE 7:**
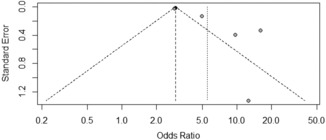
Anxiety funnel plot.

The Egger's test results revealed no publication bias associated with the factors of gender, age, marital status, chronic diseases, days served in frontline work, occupation, department of service, years of work experience, depression, received psychological assistance, worried about being infected, and degree of fear with COVID‐19 (*p* ≥ 0.05, Table 3). Years of work experience, occupation, and anxiety funnels are shown in Figures 5, 6, 7. The funnel chart reveals publication bias in anxiety, whereas publication bias in terms of years of work experience and occupation was not statistically significant.

## 7 DISCUSSION

### 7.1 Sociodemographic factors

Our research shows that women were four times more likely to experience sleep disorders than men. This is similar to the results reported in previous studies (Drake et al., 2014). The gender differences in sleep disorders may be related to biological and sociological factors. Oestrogen fluctuation is the main biological factor; it may affect the emotion‐regulating areas in the brain, making women more prone to stress and symptoms of depression and anxiety, which are also important factors casing sleep disorders (Van Eycken et al., 2020). In addition, there are also differences in family responsibilities between men and women. It has been reported that the conflict between work and family intensifies due to the sudden change in women's social roles, and therefore, women are likely to easily feel guilty about family, thus causing them a higher level of psychological distress than men (Zhan et al., 2020). Compared with the healthy healthcare workers, healthcare workers with chronic diseases, including lung disease, diabetes, hypertension, and chronic pain, are more likely to experience sleep disorders (Tang et al., 2015; Taylor et al., 2007); notably, these sleep disorders can exacerbate these chronic diseases, thus making this a vicious circle. According to the included studies, healthcare workers with poor social support during the COVID‐19 pandemic were less encouraged by family and friends to serve as frontline workers, leading to an increased likelihood of sleep disorders (Que et al., 2020). Similarly, single healthcare workers who lacked social and emotional support were more likely to experience sleep disorders than their married counterparts. Social support can reduce people's anxiety and stress, improve self‐efficacy, and indirectly improve sleep quality (Xiao et al., 2020), which may provide some ideas for preventing and managing sleep disorders in the future. Physical exercise is considered a non‐pharmacological treatment for various diseases, such as neurological diseases, metabolic diseases, and cardiovascular diseases. In our study, exercise was found to be an important factor influencing sleep quality or sleep disturbance, and it was clear that people who exercised regularly slept better. Exercising 4–8 h before bedtime can significantly reduce sleep onset latency and decrease awakenings after sleep onset. In addition, exercise can improve people's emotional state, such as reducing the prevalence of anxiety and depression, which are also important emotional factors affecting sleep. Therefore, healthcare workers can perform regular aerobic exercise, such as yoga, tai chi, and qigong, to improve sleep status after work (Luo et al., 2021).

### 7.2 Occupational factors

The development of sleep disorders, particularly insomnia, is associated with exposure to different stressors (Varela et al., 2008). Frontline healthcare workers under the stress of the COVID‐19 pandemic are facing enormous work stress and potential trauma exposure, which may lead to sleep disorders. According to reports, a healthcare worker working the night shift is 3.48 times more vulnerable to experiencing sleep disorders while fighting the COVID‐19 pandemic than a healthcare worker not working the night shift (Herrero San Martin et al., 2020). Long night shifts can disrupt homeostasis and circadian rhythms, disrupt the levels of several hormones (e.g., melatonin and cortisol), cause sleep deprivation, increase the chances of errors at work (Ballesio et al., 2021), and even increase the risk of infection (Lim et al., 2020). A new night shift schedule was tested in Wuhan, China, wherein doctors who were skilled in emergency techniques and had extensive first aid experience were only needed to work during the day, thus allowing them sufficient rest time, whereas for other doctors, their prior schedule was changed to ensure fewer night shift hours and was aligned with the already in‐practice schedule of the nurses (Zhang, Xu, et al., 2020). The results showed that this scheduling approach effectively improved the sleep quality of healthcare workers and increased the survival rate of patients. Notably, the sleep quality of healthcare workers in different departments also varied. For example, healthcare workers in isolation department and in emergency and critical care departments have more severe sleep problems. Isolation has always been the main treatment method for the COVID‐19 pandemic. Healthcare workers working in an isolated environment must wear protective equipment all the time while attending to patients confirmed or suspected as having COVID‐19. To avoid infection when taking off the protective equipment, they cannot eat or drink during working hours. This situation exhausts the spirit and body of the healthcare workers, and Zhang, Yang, et al. (2020) reported that medical staff working in an isolation environment had a 1.71‐times higher probability of experiencing insomnia. Reportedly, healthcare workers in emergency and critical care departments are also under pressure due to long‐term working environment with high workload, continuous rescue, and high mental stress, which affect their quality of sleep (Yin et al., 2021). The years of work experience and frontline work are also closely associated with the sleep disorders in healthcare workers. Fewer working years represent less work experience (Wang, Hu, et al., 2020; Wang, Huang, et al., 2020; Wang, Liu, et al., 2020; Wang, Ren, et al., 2020) and translate into poor readiness to cope with the crisis (Zhou et al., 2020) and higher chances of developing sleep disorders.

### 7.3 Psychological factors

During the COVID‐19 pandemic, anxiety and depression are the negative emotions frequently encountered by healthcare workers, and they are also risk factors for sleep disorders. Healthcare workers with anxiety or depression often face difficulty falling asleep or waking up repeatedly after falling asleep, thus preventing them from entering deep sleep. Sleep deprivation can lead to daytime fatigue, loss of motivation, and increased risk of mistakes at work, which in turn exacerbates these negative emotions, leading to a vicious cycle (Ballesio et al., 2021). As the number of COVID‐19 infections rises, the work pressure and psychological pressure of healthcare workers also increase. Physiological studies have found that frequently waking up and high‐frequency EEG power during non‐rapid eye movement contributed to increased fear and stress, leading to the development of short‐term insomnia (Cano et al., 2008). Fatigue was also identified as a factor affecting sleep quality in this study, and compared to people without fatigue, those with fatigue were more likely to experience symptoms of insomnia and sleepiness during the day (Kim et al., 2019). Previous studies have shown that fatigue is directly affected by working hours, workload, anxiety, and work satisfaction (Chen et al., 2017). Therefore, Chinese hospitals have tried to alleviate or minimize fatigue by implementing 6‐h shifts, reasonable human resource allocation, encouraging nurses to communicate with family and friends, increasing wage subsidies, and promoting nurses' work satisfaction (Zhan et al., 2020). Research has found that healthcare workers' concerns about infection were associated with concerns about personal and family health (Nickell et al., 2004), and the anxiety emanating from these concerns may harm their sleep quality (Kirwan et al., 2017). Consequently, healthcare workers who were worried about being infected were more likely to have sleep disorders.

## 8 CONCLUSIONS

During the COVID‐19 pandemic, the incidence of sleep disorders in healthcare workers has increased and translated into poor sleep quality, particularly in frontline healthcare workers who are in direct contact with patients confirmed or suspected as having COVID‐19. The occurrence of these sleep disorders is significantly associated with sociodemographic factors (gender, marital status, chronic diseases, insomnia history, exercise, and social support), occupational factors (frontline work, days served in frontline work, department of service, night shift, and years of work experience), and psychological factors (anxiety, depression, stress, received psychological assistance, worried about being infected, and degree of fear with COVID‐19). The results of this study can help medical institutions better understand healthcare workers' sleep disorders during the COVID‐19 pandemic and provide corresponding measures. It is recommended to provide more care for female and single healthcare workers and for healthcare workers with poor social support. Supportive measures include reasonable arrangements for night shifts, regular psychological counselling for frontline healthcare workers, and conducting scheduled aerobic exercises daily to improve the sleep quality and mental health of healthcare workers.

## 9 LIMITATIONS

This study has several limitations. First, we only included the influencing factors mentioned in ≥2 studies and did not include the influencing factors mentioned in only one literature. Second, not all studies provided information on some of the factors that may influence the OR and 95% CI, such as professional title and health status, and we did not pool the effect size, which may have affected the reliability of the results to a certain extent. Third, the division criteria of some influencing factors are different, and therefore, it is difficult to draw more specific conclusions. Finally, although we have implemented a comprehensive search of the studies, the language of the included studies was limited to English and Chinese, which may have caused publication bias.

## AUTHOR CONTRIBUTIONS

Q.L. conceptualized the study. Q.L. and W.Z. wrote the first version. Y.K reviewed and revised it critically for important intellectual content. Q.L. and S.C. searched the database and collected the data. B.X., F.Z., and X.S. evaluated the quality of literature. Q.L., S.C and Z.Q conducted meta‐analyses. Q.L and W.Z contributed equally to this study.

## FUNDING INFORMATION

This study was supported by Development and application of functional protective gear for medical personnel based on biosafety (20SWAQK48) and Fujian Science and Technology Planning Project (2020Y0080) in China.

## CONFLICT OF INTEREST STATEMENT

The authors declare that they have no conflict of interest.

## Data Availability

The data used to support the findings of this study are available from the corresponding author on reasonable request.
